# Stopping chromosomes from breaking bad

**DOI:** 10.7554/eLife.27292

**Published:** 2017-05-09

**Authors:** Rima Sandhu, G Valentin Börner

**Affiliations:** Center for Gene Regulation in Health and Disease, Cleveland State University, Cleveland, United States; Center for Gene Regulation in Health and Disease, Cleveland State University, Cleveland, United Statesg.boerner@csuohio.edu

**Keywords:** Polo-like kinase, double-strand breaks, SYP-4, synaptonemal complex, meiosis, *C. elegans*

## Abstract

The scaffolding that holds chromosome pairs together plays a key role in limiting the levels of double-strand breaks.

**Related research article** Nadarajan S, Lambert TJ, Altendorfer E, Gao J, Blower MD, Waters JC, Colaiácovo MP. 2017. Polo-like kinase-dependent phosphorylation of the synaptonemal complex protein SYP-4 regulates double-strand break formation through a negative feedback loop. *eLife* **6**:e23437. doi: 10.7554/eLife.23437

When is the right time for an organism to terminate a developmental process? This is a question that is relevant to most levels of biological organization: when should the development of an organ, such as the heart, stop? When has enough of a subcellular structure, such as the endoplasmic reticulum, been formed? This question is especially relevant when a failure to stop a process could damage the organism. Such processes include the programmed induction of double-strand breaks, which form in large numbers along chromosomes at the onset of meiosis (Keeney et al., 2014).

Double-strand breaks have a crucial role in meiosis because some of them become “crossovers” between homologous chromosomes (homologs). Successful meiosis requires the formation of at least one such crossover along each pair of homologs. Yet, the formation of double-strand breaks must also be stopped in a timely manner to prevent segments of chromosomes from being lost. Now, in eLife, Monica Colaiácovo and colleagues at Harvard Medical School and Massachusetts General Hospital – including Saravanapriah Nadarajan as first author – report that designating a double-strand break to be a future crossover shuts down the formation of further breaks (Nadarajan et al., 2017). Intriguingly, the exact molecular architecture of the synaptonemal complex, the scaffolding that connects homologs along their length before they separate during meiosis I, appears to play a key role in this process.

The work by Nadarajan et al. belongs to a flurry of papers that have recently challenged long-standing ideas about the structure of the synaptonemal complex (Machovina et al., 2016; Rog et al., 2017; Pattabiraman et al., 2017). At any given time, the central element of this complex appears as a static scaffold comprised of regularly spaced transverse filaments. When the movement of individual filament proteins is tracked, it is evident that they are continuously unloaded and replaced by new molecules. This rapid turnover slows down only when a subset of double-strand breaks has been designated as sites of future crossovers.

SYP-4 is one of four transverse filament proteins that make up the central element of the synaptonemal complex in the nematode worm *Caenorhabditis elegans* (Smolikov et al., 2009). Nadarajan et al. now show that phosphorylation at a particular site (serine 269) on SYP-4 has two consequences. First, additional double-strand break formation is stopped. Accordingly, the elimination of SYP-4 phosphorylation results in the continued appearance of breaks even after the obligatory crossover site per homolog pair has been designated. In other words, there is a negative feedback loop that ensures that a site committed to become a crossover prevents further double-strand breaks from forming.

Second, phosphorylation also results in the stabilization of the structure of the central region of the synaptonemal complex. Accordingly, without SYP-4 phosphorylation, rapid turnover of transverse filament proteins in the central element continues even though the required number of crossovers has been reached. Nadarajan et al. argue that a failure to stabilize the synaptonemal complex in a timely manner allows the formation of double-strand breaks to continue. They exclude the opposite effect (that excessive breaks destabilize the synaptonemal complex) because double-strand break levels are not generally correlated with the dynamics of this structure (Pattabiraman et al., 2017).

Some intriguing parallels exist between the mechanism identified in *C. elegans* and two mechanisms identified in budding yeast that also prevent formation of excessive double-strand breaks (Keeney et al., 2014). In the first case, excessive break formation is prevented via phosphorylation of yeast Rec114, a widely conserved accessory protein that is required for double-strand break formation (Carballo et al., 2013). In the second case, additional breaks are suppressed by the phosphorylation of a yeast transverse filament protein called Zip1 (Thacker et al., 2014; Chen et al., 2015). Like SYP-4, the phosphorylation of Rec114 and Zip1 depend on the formation of double-strand breaks, although further maturation of breaks into crossovers does not appear to be required (Carballo et al., 2013; Chen et al., 2015).

Additional commonalities between the two systems include the fact that phosphorylation triggers structural changes between and along homologs. Accordingly, phosphorylation results in a shift of Rec114 away from its initial double-strand break association, whereas Zip1 phosphorylation triggers close homolog juxtaposition via the synaptonemal complex (Carballo et al., 2013; Chen et al., 2015). At the same time, it is presently unclear whether these changes are mediated in different organisms by the same kinases (i.e. the enzymes that phosphorylate proteins).

The discovery that the molecular architecture of the synaptonemal complex plays a central role in terminating the formation of double-strand breaks opens up intriguing avenues for further investigation. Components of the central element of the synaptonemal complex are normally not required for double-strand break formation. How then does stabilizing the central element inactivate the formation of breaks? One possibility is that it alters how easily the double-strand break machinery can access its targets along chromosomes. Alternatively, stabilization of the synaptonemal complex may interfere with initial break processing thereby allowing the Spo11 topoisomerase that normally induces meiotic breaks to shift its activity to the backward reaction and bind breaks back together (Keeney et al., 2014; Prieler et al., 2005).

We also need a better understanding of the relationship between the mechanisms that prevent excess double-strand break formation and the mechanisms that ensure that enough breaks are formed. In *C. elegans*, both depend on polo-like kinases and crossover designation factors (Machovina et al., 2016; Pattabiraman et al., 2017). Thus, positive and negative feedback loops that either enhance or limit the formation of double-strand breaks share multiple components. Nadarajan et al. have identified the phosphorylation of SYP-4 as a critical switch for terminating break formation. It will be fascinating to identify the other factors that help a cell to decide whether formation of additional double-strand breaks should be reduced or enhanced.
